# Patterns and predictors of analgesic use in pregnancy: a longitudinal drug utilization study with special focus on women with migraine

**DOI:** 10.1186/s12884-017-1399-0

**Published:** 2017-07-14

**Authors:** Gerd-Marie Eskerud Harris, Mollie Wood, Malin Eberhard-Gran, Christofer Lundqvist, Hedvig Nordeng

**Affiliations:** 1 Pharmacoepidemiology & Drug Safety Research Group, School of Pharmacy, Faculty of Mathematics and Natural Sciences, University of Oslo, P.O. Box. 1068, Blindern, 0316 Oslo, Norway; 2 Department of Child Health, National Institute of Public Health, Oslo, Norway; 3 Health Services Research, Research Department, Akershus University Hospital and University of Oslo, Campus Ahus, Lørenskog, Norway

**Keywords:** Pregnancy, Drug utilization, Migraine, Analgesics, Predictors

## Abstract

**Background:**

Few studies have investigated the drug utilization patterns and factors predicting drug use in pregnant women with migraine. This longitudinal drug utilization study aimed to describe patterns of analgesic use in a sample of Norwegian pregnant women according to their migraine history, and to identify predictors for analgesic use among these women.

**Methods:**

Pregnant women giving birth at Akershus University Hospital between 2008 and 2010 were recruited at ultrasound examination in gestational week 17. Data were collected by questionnaires in gestational weeks 17 and 32, and at 8 weeks postpartum, and linked to birth records. Women were grouped into four categories according to migraine history: no migraine history, previous migraine history, recent migraine history (within 1 year prior to pregnancy) and migraine in pregnancy. Patterns of use of analgesics were analyzed descriptively. Multivariable logistic regression was used to identify factors predicting analgesic use.

**Results:**

Out of 1981 women, 5.0% reported having migraine in pregnancy, 13.2% had a recent history of migraine, 11.5% had a previous history of migraine, and 68.8% reported no history of migraine. Analgesic use declined during pregnancy. Many women switched from triptans and non-steroidal anti-inflammatory drugs to paracetamol, which constituted most of the analgesic use. Factors associated with analgesic use included recent migraine history (OR 1.6, 95% CI 1.2–2.2), more severe headache intensity (OR 1.3, 95% CI 1.3–1.4), smoking (OR 1.9, 95% CI 1.1–3.3) and multiparity (OR 1.4, 95% CI 1.1–1.7).

**Conclusions:**

Women with migraine stop or switch medications during pregnancy. Analgesic use in pregnancy is affected by migraine characteristics and intensity, and also by socio-demographic factors. Clinicians should bear this in mind when giving advice on adequate management of migraine in pregnancy and safe analgesic use.

**Electronic supplementary material:**

The online version of this article (doi:10.1186/s12884-017-1399-0) contains supplementary material, which is available to authorized users.

## Background

Migraine affects approximately 20% of women of reproductive age [1]. Many women suffering from migraine experience an improvement in migraine symptoms during pregnancy, and about one third report complete remission [2, 3]. However, pharmacotherapy is still necessary for many pregnant women with migraine. Analgesics, including triptans, non-steroidal anti-inflammatory drugs (NSAIDs), paracetamol and opioids, are commonly used to treat migraine, and paracetamol is recommended as first choice during pregnancy [4, 5].

Medical treatment of pregnant women is a challenge, and a balance between benefit for the mother and risk to the child must be maintained. The risk to the child is often overestimated, and will influence the decision to use a drug in pregnancy [6, 7]. Socio-demographic characteristics and lifestyle factors may also impact medication use, but the results from previous studies are inconsistent [8–12]. Prevalence and patterns of medication use have been shown to vary between countries [9, 13]. In a large multinational study conducted in 2011–2012, 81% of all pregnant women used medications. Analgesics were the most common class of drugs, used by 56% of the women (38% in first trimester, 44% in second, 36% in third) [8].

Few studies have investigated drug utilization patterns and factors predicting drug use in pregnant women with migraine. One population-based study found that 73% of pregnant women with migraine used anti-migraine drugs, mostly non-narcotic drugs (54%) and triptans (25%) [14]. This study also found a positive association between use of anti-migraine drugs and high body mass index, little sleep and being on sick-leave. Multiparous women were less likely to use triptans, but more likely to use other anti-migraine medications [14]. In another registry-based study, pregnant women using anti-migraine drugs (mainly sumatriptan) were older and more often primiparous than women not using such drugs [15]. A recently published cross-sectional study found no associations between anti-migraine medication use and migraine-related, socio-demographic or lifestyle factors, when adjusting for migraine severity [16].

In order to provide optimal treatment and counselling to women with migraine who are pregnant or are planning to become pregnant, knowledge about drug utilization patterns and maternal factors affecting these patterns are necessary. This study aims to describe patterns of analgesic use in a sample of Norwegian pregnant women according to their migraine history, and to identify predictors for analgesic use among these women. Based on previous studies, we hypothesized that the drug utilization patterns would decrease in pregnancy and that severity of migraine would be more important than socio-demographic, lifestyle and other medical factors for use of analgesics among pregnant women.

## Methods

### Study sample and design

The current study uses data from the Akershus Birth Cohort Study (ABC study), which targeted all pregnant women scheduled to deliver at Akershus University Hospital (Ahus) in Norway. The hospital serves a population of approximately 400,000 individuals from both urban and rural surroundings, and had an average birth rate of 3500 births each year during the study period.

Pregnant women were recruited between November 2008 and April 2010 by a trained midwife at ultrasound examination in gestational week 17–20. This examination is offered to all pregnant women free of charge as part of the public antenatal care program in Norway. There were no exclusion criteria other than not being able to complete a questionnaire in Norwegian. Of the 4814 pregnant women invited to participate, 4623 women were included in the study (96.0%).

**Fig. 1:**
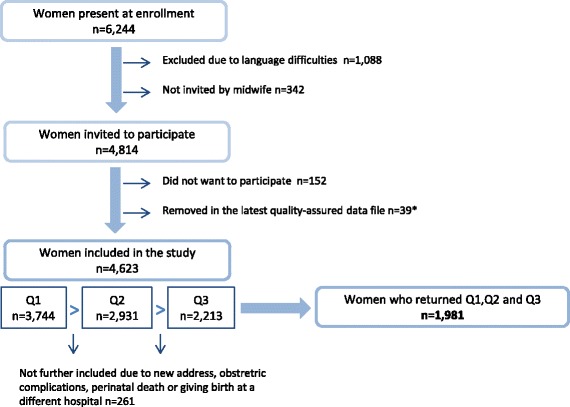
The Akershus Birth Cohort: overview of inclusion, response rates and the study sample. *Note that the sample sizes may deviate somewhat from previous publications based on this data material due to small changes in the latest quality-assured data file released for research

Data were collected by self-completed questionnaires in gestational weeks 17 (Q1) and 32 (Q2), and at 8 weeks postpartum (Q3), and thereafter linked to the hospital birth records. The response rates were 81.0% (3744 of 4623), 81.1% (2931 of 3613) and 79.0% (2213 of 2801), respectively. For the current study, the final study sample consisted of 1981 women who completed all three questionnaires, representing 42.9% of those included. Women included in our study sample were older, less often smokers, more often married or cohabiting, had higher education and higher headache intensity, compared to those in the full cohort (Additional file 1: Table S1). An overview of inclusion, response rates and the study sample is presented in Fig. 1.

### Maternal characteristics

**Fig. 2:**
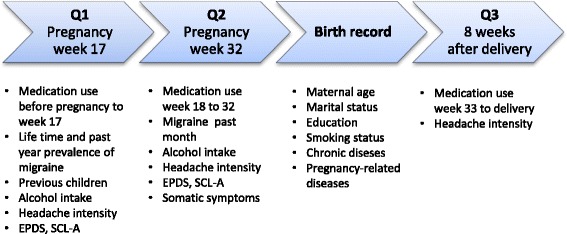
The Akershus Birth Cohort: overview of the relevant variables measured in the questionnaires and birth record

An overview of the sources of the relevant variables describing maternal characteristics is presented in Fig. 2.

#### Migraine characteristics

Migraine was self-reported in the first and second questionnaires, which include specific questions about lifetime prevalence of migraine (Q1; “Have you ever had migraine?”), and migraine frequency in the past month (Q2; “How many days have you had migraine within the past month?”). This enabled us to group women into four mutually exclusive categories according to migraine pattern: no migraine history (“never had migraine”), previous history of migraine (“have had migraine, but not during the past year”), recent history of migraine (within 1 year prior to pregnancy) (“have had migraine during the past year”), and migraine in pregnancy (one or more days with migraine within the past month during the last part of pregnancy).

Headache intensity was measured in the first and second questionnaire by a numeric rating scale from 0 to 10 where 0 is no pain at all and 10 is the strongest pain imaginable, categorized as low (0–3), moderate (4–6) or high (7–10).

#### Analgesic use

Women were specifically asked about use of drugs within seven categories – drugs for headache, migraine, non-headache pain, insomnia, anxiety, depression and other psychotropic medications. For each medication group, the women could tick yes or no as to whether she used such a drug, and fill in the name of the medication. The three questionnaires cover different periods of use – four months before pregnancy and beginning of pregnancy until week 17 (Q1), week 18 to 32 (Q2), and the last part of pregnancy from week 33 forward (Q3).

Drug exposure was coded in groups based on the Anatomical Therapeutic Chemical (ATC) Classification System [17]. We defined use of analgesics as use of paracetamol (N02BE01), NSAIDs (M01A), triptans (N02CC) or opioids (N02A) reported as used for either headache or migraine. Variables were created for the pre-pregnancy period, pregnancy overall, and the time-specific periods in pregnancy covered by Q1, Q2 and Q3 – for analgesics overall and for the different substances.

#### Socio-demographic characteristics, lifestyle factors and comorbidity

Socio-demographic and lifestyle characteristics were categorized as presented in Table 1.

Having symptoms of depression or anxiety was defined as having a score ≥ 13 on the ten-item self-rating Edinburgh Postnatal Depression Scale (EPDS) [18] and/or a score ≥ 18 on the first ten items (SCL-anxiety) of the 25-item Hopkins Symptoms Checklist (SCL-25) [19, 20], in either Q1 or Q2. Both instruments are widely used and validated as tools for detecting symptoms of depression and anxiety in pregnancy [21–23].

Chronic diseases recorded in the birth records include heart disease, chronic hypertension, chronic kidney disease, recurring urinary tract infections, gynecological conditions, asthma, allergy, epilepsy, rheumatoid arthritis, diabetes, genetic disorders and psychiatric disorders. These were coded as no diseases, one disease or two or more diseases. Pregnancy-related diseases include gestational diabetes, gestational hypertension, preeclampsia, eclampsia and hyperemesis, coded as no pregnancy-related diseases or one or more pregnancy-related disease(s). A variable for somatic symptoms was also created, based on a somatic symptom scale in Q2 derived from the Primary care Evaluation on Mental Disorders (PRIME-MD) [24], which included (yes/no): stomach pain, back pain, pain in arms/legs/joints, menstrual pain or problems, pain or problems during sexual intercourse, headache, chest pain, dizziness, fainting spells, feeling your heart pound or race, shortness of breath, constipation/diarrhea/indigestion, feeling tired or having low energy, and having trouble sleeping. They were coded no somatic symptoms, 1–2 somatic symptoms, 3–4 somatic symptoms, and 5 or more somatic symptoms.

### Statistical analyses

Patterns of analgesic use were analyzed descriptively for the 1981 women present at all three time points. Possible predictors for analgesic use were identified using multivariable logistic regression, with the outcome variable defined as use of analgesics at least once during pregnancy. The variables listed in Table 1 were considered as potential predictors for analgesic use. The selection of variables to be included in the potential predictor sets was based on results from previous studies, as well as the results from exploratory data analysis. Possible high inter-correlations among the independent variables were checked for, using multiple regression analysis and ensuring that the tolerance values for collinearity statistics were adequate (>0.1).​ The covariates were fitted in multivariable logistic regression models using area under Receiver Operating Characteristics (ROC) curves and likelihood ratio tests in a backwards selection process. The analyses were restricted to complete cases. All the covariates in Table 1 were retained in the final multivariable model. Maternal age, somatic symptoms and headache intensity were used as continuous variables. The Hosmer-Lemeshow test was used to assess goodness-of-fit of the model, and *p* > 0.05 was considered robust [25]. StataMP release 14 was used in all statistical analyses [26].

## Results

### Characteristics of the study sample and sub-samples

Of the total study sample of 1981 pregnant women, 100 (5.0%) reported having migraine in pregnancy, 262 (13.2%) had a recent history of migraine (within the past year prior to pregnancy), 227 (11.5%) had a previous history of migraine, and 1362 (68.8%) reported having no history of migraine. Characteristics of the women in the different groups are presented in Table 1. The mean age was 31.3 years (standard deviation 4.6, range 18.8–45.5 years). Age, parity, marital status, education, alcohol use, chronic diseases, and pregnancy related diseases were equally distributed across these migraine-related subgroups of women. Women with migraine were more likely to smoke and to have symptoms of depression or anxiety. They also had more somatic symptoms, higher headache intensity, and used more analgesics in pregnancy.

**Table 1:** Overview of maternal characteristics and analgesic use in pregnancy in the total study sample and in sub-samples according to migraine pattern

	Study sample	Sub-samples according to migraine pattern			
	Total number of women	No history of migraine	Previous history of migraine	Recent history of migraine	Migraine in pregnancy
	*n* = 1981 *n* (% of *n*)	*n* = 1362 *n* (% of *n*)	*n* = 227 *n* (% of *n*)	*n* = 262 *n* (% of *n*)	*n* = 100 *n* (% of *n*)
Maternal age at delivery					
< 25	165 (8.3)	119 (8.7)	12 (5.3)	16 (6.1)	15 (15.0)
25–30	588 (29.7)	403 (29.6)	64 (28.2)	79 (30.2)	29 (29.0)
31–35	768 (38.8)	529 (38.8)	95 (41.9)	100 (38.2)	38 (38.0)
> 35	446 (22.5)	303 (22.2)	56 (24.7)	62 (23.7)	17 (17.0)
Parity					
First time mother	988 (49.9)	669 (49.1)	102 (44.9)	148 (56.5)	53 (53.0)
≥ 1 previous child	993 (50.1)	693 (50.9)	125 (55.1)	114 (43.5)	47 (47.0)
Marital status					
Married/cohabiting	1910 (96.4)	1318 (96.8)	222 (97.8)	246 (93.9)	94 (94.0)
Single/divorced/separated	44 (2.2)	28 (2.1)	5 (2.2)	7 (2.7)	4 (4.0)
Education					
College/university	1294 (65.3)	905 (66.4)	146 (64.3)	160 (61.1)	61 (61.0)
Primary/secondary school	606 (30.6)	406 (29.8)	69 (30.4)	86 (32.8)	37 (37.0)
Smoking at time of delivery					
No	1841 (92.9)	1280 (94.0)	211 (93.0)	235 (89.7)	87 (87.0)
Yes	76 (3.8)	42 (3.1)	11 (4.8)	14 (5.3)	8 (8.0)
Alcohol in pregnancy					
No	1866 (94.2)	1282 (94.1)	210 (92.5)	255 (97.3)	93 (93.0)
Yes	82 (4.1)	56 (4.1)	11 (4.8)	7 (2.7)	6 (6.0)
Symptoms of depression or anxiety in pregnancy					
No	1684 (85.0)	1184 (86.9)	192 (84.6)	218 (83.2)	68 (68.0)
Yes	291 (14.7)	172 (12.6)	35 (15.4)	44 (16.8)	32 (32.0)
Chronic diseases					
None	831 (41.9)	590 (43.3)	92 (40.5)	102 (38.9)	34 (34.0)
1 disease	747 (37.7)	506 (37.2)	94 (41.4)	95 (36.3)	40 (40.0)
≥ 2 diseases	392 (19.8)	260 (19.1)	41 (18.1)	61 (23.3)	25 (25.0)
Pregnancy related diseases					
None	1777 (89.7)	1221 (89.6)	212 (93.4)	229 (87.4)	87 (87.0)
≥ 1 disease	191 (9.6)	134 (9.8)	15 (6.6)	29 (11.1)	11 (11.0)
Somatic symptoms					
None	182 (9.2)	139 (10.2)	20 (8.8)	20 (7.6)	1 (1.0)
1–2 symptoms	729 (36.8)	533 (39.1)	80 (35.2)	83 (31.7)	20 (20.0)
3–4 symptoms	658 (33.2)	430 (31.6)	84 (37.0)	93 (35.5)	41 (41.0)
≥ 5 symptoms	412 (20.8)	260 (19.1)	43 (18.9)	66 (25.2)	38 (38.0)
Headache intensity					
Low	741 (37.4)	627 (46.0)	60 (26.4)	35 (13.4)	9 (9.0)
Moderate	979 (49.4)	600 (44.1)	143 (63.0)	179 (68.3)	43 (43.0)
High	183 (9.2)	61 (4.5)	22 (9.7)	48 (18.3)	48 (48.0)
Analgesics in pregnancy					
No	1004 (50.7)	766 (56.2)	107 (47.1)	90 (34.4)	28 (28.0)
Yes	977 (49.3)	596 (43.8)	120 (52.9)	172 (65.6)	72 (72.0)

Numbers do not add up to the total in each group due to missing values: migraine history *n* = 30 (1.5%), maternal age *n* = 14 (0.7%), marital status *n* = 27 (1.4%), education *n* = 81 (4.1%), smoking *n* = 64 (3.2%), alcohol *n* = 33 (1.7%), symptoms of depression/anxiety *n* = 6 (0.3%), headache intensity *n* = 78 (3.9%), chronic diseases *n* = 11 (0.6%), pregnancy related diseases *n* = 13 (0.7%)

### Patterns of analgesic use

**Fig. 3:**
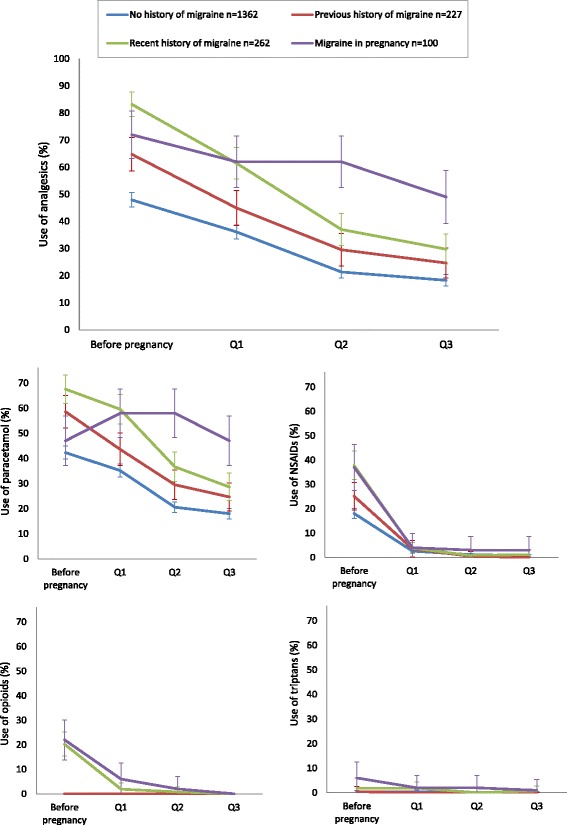
Patterns of total analgesic use and use of specific analgesics before and during pregnancy among women with no migraine history, previous history of migraine, recent history of migraine (within 1 year prior to pregnancy) and migraine in the past month (% users in each group with 95% confidence intervals). Analgesics include paracetamol, NSAIDs, opioids and triptans, used for either migraine or headache

Use of analgesics for headache or migraine was reported by a total of 977 women (49.3%) in pregnancy, compared to 1107 (55.9%) before pregnancy. Among women who reported migraine in pregnancy, 72.0% used analgesics during pregnancy. The patterns of analgesic use overall and of specific analgesics are shown in Fig. 3. Analgesic use declined for all groups of women, both at the beginning of pregnancy and during pregnancy. The medication groups NSAIDs, opioids, and triptans had a prominent drop in pregnancy compared to before pregnancy, and were used by less than 6.0% in pregnancy. Paracetamol constituted most of the analgesic use in pregnancy in all groups of women. It was also the most common medication before pregnancy, followed by NSAIDs, triptans (for women with migraine) and opioids. Of those women using triptans and NSAIDs prior to pregnancy, 64.0% of the triptan users and 69.9% of the NSAIDs users switched to paracetamol in pregnancy (alone or in combination with other analgesics). One third of women using analgesics prior to pregnancy discontinued in pregnancy (Additional file 2: Figure S1).

Almost half of the women with migraine in pregnancy had high headache intensity (48.0%). Of these, 18.8% did not use any analgesics, 66.7% used paracetamol alone and 14.5% used paracetamol in combination with other analgesics (Additional file 3: Figure S2).

### Predictors of analgesic use

Results from the logistic regression analyses are given in Table 2. Factors positively associated with analgesic use included having a recent migraine history (Adj. OR 1.59, 95% CI 1.16–2.18), headache intensity (Adj. OR 1.33, 95% CI 1.25–1.43), smoking (Adj. OR 1.89, 95% CI 1.09–3.28), multiparity (Adj. OR 1.38, 95% CI 1.11–1.72), somatic symptoms (Adj. OR 1.08, 95% CI 1.03–1.14) and having two or more chronic diseases (Adj. OR 1.36, 95% CI 1.03–1.79). Having migraine in pregnancy and drinking alcohol in pregnancy were borderline significant.

**Table 2:** Overview of maternal characteristics, prevalence of analgesic use and associations between maternal characteristics and analgesic use

	Study sample	Prevalence of analgesic use	Associations between possible predictors and analgesic use	
	*n*	*n* (% of *n*)	Crude OR (95% CI)	Adjusted OR (95% CI)
Maternal age at delivery				
< 25	165	77 (46.7)	1.00 (0.98–1.02)	0.99 (0.97–1.01)
25–30	588	304 (51.7)		
31–35	768	385 (50.1)		
> 35	446	205 (46.0)		
Parity				
First time mother	988	450 (45.6)	1	1
≥ 1 previous child	993	527 (53.1)	**1.37 (1.13–1.65)**	**1.38(1.11–1.72)**
Marital status				
Married/cohabiting	1910	947 (49.6)	1	1
Single/divorced/separated	44	19 (43.2)	0.74 (0.40–1.39)	0.62 (0.32–1.21)
Education				
College/university	1294	609 (47.1)	1	1
Primary/secondary school	606	332 (54.8)	**1.27 (1.04–1.56)**	1.07 (0.85–1.35)
Smoking at time of delivery				
No	1841	893 (48.5)	1	1
Yes	76	52 (68.4)	**2.25 (1.35–3.75)**	**1.89 (1.09–3.28)**
Alcohol in pregnancy				
No	1866	918 (49.2)	1	1
Yes	82	46 (56.1)	1.41 (0.87–2.28)	1.60 (0.95–2.69)
Symptoms of depression or anxiety in pregnancy				
No	1684	806 (47.9)	1	1
Yes	291	170 (58.4)	**1.41 (1.07–1.84)**	0.86 (0.63–1.18)
Chronic diseases				
None	831	381 (45.8)	1	1
1 disease	747	368 (49.3)	1.10 (0.89–1.35)	1.08 (0.86–1.35)
≥ 2 diseases	392	223 (56.9)	**1.45 (1.12–1.88)**	**1.36 (1.03–1.79)**
Pregnancy related diseases				
None	1777	878 (49.4)	1	1
≥ 1 disease	191	93 (48.7)	0.91 (0.66–1.24)	0.86 (0–62–1.21)
Somatic symptoms				
None	182	59 (32.4)	**1.15 (1.10–1.20)**	**1.08 (1.03–1.14)**
1–2 symptoms	729	329 (45.1)		
3–4 symptoms	658	339 (51.5)		
≥ 5 symptoms	412	250 (60.7)		
Headache intensity				
Low	741	257 (34.7)	**1.42 (1.33–1.50)**	**1.33 (1.25–1.43)**
Moderate	979	593 (60.6)		
High	183	120 (65.6)		
Migraine pattern				
No history	1362	596 (43.8)	1	1
Previous history	227	120 (52.9)	**1.36 (1.01–1.83)**	1.09 (0.80–1.49)
Recent history prior to pregnancy	262	172 (65.6)	**2.33 (1.74–3.13)**	**1.59 (1.16–2.18)**
Migraine in pregnancy	100	72 (72.0)	**3.35 (2.07–5.41)**	1.56 (0.93–2.63)

Numbers do not add up to the total due to missing values (<5.0% for all variables). Complete cases *n* = 1721. Area under ROC curve = 0.69. Adj. ORs are adjusted for all covariates in the table. Significant associations are marked in bold. Maternal age, somatic symptoms and headache intensity were analyzed as continuous variables. Analgesics include paracetamol, NSAIDs, opioids and triptans, used for either headache or migraine

## Discussion

### Main findings

Total analgesic use declined at the beginning of pregnancy and continued to decline throughout pregnancy. Analgesics that are not recommended or have limited safety documentation were drastically reduced, and paracetamol was by far the most commonly used analgesic. For the subgroup of women with active migraine, an increase in use of paracetamol was seen in pregnancy, suggesting that women on strong pain medications switched to paracetamol during pregnancy. Both migraine itself and headache intensity were associated with an increased likelihood of analgesic use, as were somatic symptoms, chronic diseases, smoking and parity.

### Strengths and limitations

The ABC study had a high response rate and included women attending routine antenatal care. However, only Norwegian speaking women were included, which could limit the generalizability of the results, as other ethnic groups may have different attitudes and traditions towards medication use in pregnancy [27]. For the present study, we required women to have responded to all three questionnaires, representing only 42.9% of all study participants. A comparison of these women with the full cohort population and the general birthing population of Norway (Additional file 1: Table S1), indicate that the women in our sample, like in most questionnaire-based studies, were possibly of higher socioeconomic status. This may limit the generalizability of our findings and might have affected our results, especially prevalence and patterns of analgesic use. Associations are less likely to be affected by selection bias than prevalence estimates [28, 29]. However, this should be taken into consideration when interpreting the results.

Both migraine and analgesic use is self-reported, and the nature of this type of study has both strengths and limitations. Self-reported medication use allows us to assess use of over-the-counter (OTC) analgesics as well as prescription analgesic use, which would not be possible using prescription registry or administrative data. However, some women might not remember or might not want to report use of medications. Previous research has found that for self-reported analgesic use, sensitivity may be low, but specificity is generally quite high [30]. Even though specific indications for use were named in the questionnaires to promote reporting, there could still be underreporting of analgesic use, particularly OTC analgesics. This would bias our effect estimates towards the null, and might lead to some factors not being identified as predictors when they should be, or underestimation of the strength of some predictors*.* Because we combined the categories for headache and migraine, we do not know which indication the drugs were actually used for; even in the active migraine group, drugs could have been used for non-migraine headache. This is particularity relevant for paracetamol, as it is widely used for all types of headache and is the recommended analgesic during pregnancy; therefore its use for migraine could be overestimated in our study [31].

Relying on self-reported diagnoses depends on the woman’s own perception of her medical condition, which might lead to misclassification. However, the agreement between self-reported lifetime prevalence of migraine (as asked in the questionnaire), and migraine diagnosis as classified by the International Headache Society has been shown to be good, with a kappa value of 0.81 [32]. The validity of self-reported migraine has also been demonstrated in other studies [33, 34].

Finally, we were not able to do sub-analyses on specific analgesics, as very few participants used NSAIDs, opioids or triptans in pregnancy. It could be that other factors are driving use of stronger pain medication compared to paracetamol.

### Interpretation

We found a marked decrease in analgesic use in pregnancy, and also throughout pregnancy. We know that many pregnancies are not planned, so a number of women might have been taking drugs before they discovered they were pregnant [35]. On the other hand, the first part of pregnancy is the most vulnerable period with regards to malformations, so we would expect women to be more cautious about medication use early in pregnancy [31].

The patterns were clearly different for the different medication groups. NSAIDs and triptans, which are generally not recommended, or recommended to be used with caution in pregnancy, were drastically reduced. However, despite the reduction in NSAID use, some women did persist in using NSAIDs early in pregnancy, possibly owing to their availability over the counter. The use of OTC drugs in pregnancy is common, which has been seen also for migraine patients [15, 36]. Opioids are only recommended for limited use and have no licensed indication for migraine, which was reflected by little use both before and during pregnancy. The clear shift to paracetamol is in line with recommendations for treatment of mild to moderate migraine during pregnancy [4], although recent concerns have been raised regarding use of paracetamol in pregnancy due to possible effects on neurodevelopment [37, 38].

Migraine is often improved in pregnancy [2, 3], and many pregnant women may therefore not require migraine therapy, which could explain the decrease in analgesic use for the group with recent migraine prior to pregnancy. For women with active migraine in pregnancy, the pattern was different; overall analgesic use decreased slightly and paracetamol increased in the beginning of pregnancy. These women could also have experienced an improvement in migraine symptoms, and thereby have adequate effect from paracetamol. Another explanation could be reluctance to use stronger analgesics, and caution among physicians to prescribe drugs that are not sufficiently documented in pregnancy.

Few studies have investigated the patterns of use of headache and migraine medications in pregnancy. A Norwegian cohort study identified 3000 women with migraine before and during pregnancy and found no change in the extent or type of medications used before and during pregnancy [14]. Although not directly comparable with our study, our findings show the opposite trend, with a distinct switch to less effective, but more established drugs in pregnancy. The same trend was observed in a cross-sectional study including 400 pregnant or lactating women with migraine [16].

A considerable number of women reported high headache intensity, and many of them did not use any analgesics, while the remaining mainly used paracetamol. This suggests that there are women who are not optimally treated. This finding was also reported in another study, where less than one third of the women considered their migraine to be optimally treated [16]. For women with insufficient relief from paracetamol or with severe migraine, limited use of triptans should be considered and recognized as an alternative by prescribers. Results from studies on triptan safety in pregnancy are generally reassuring and suggest that sporadic use of sumatriptan is probably safe, although the data are limited for other triptans [4]. While it is necessary to exercise caution when using pharmacotherapy during pregnancy, untreated or inadequately managed severe migraine may seriously impact a woman’s well-being, and might even pose a risk to both mother and child. In fact, several studies have suggested an association between active migraine and hypertensive diseases in pregnancy [39–42], which is a risk factor for preterm birth, low birthweight and placental abruption [43]. Moreover, the switch to less effective migraine medications such as paracetamol could impose an increased risk of analgesic induced headache [44].

We found several factors predicting analgesic use, some of them not directly related to the disease, which illustrates the need for and importance of information and advice on safe analgesic use and migraine management in pregnancy. This should be recognized by physicians as well as midwives, pharmacists, and other health care personnel communicating with pregnant women. The fact that smokers were more likely to use analgesics for headache or migraine can be due to a less restrictive attitude towards medication use in pregnancy, or addictive behavior [45]. Mothers with previous pregnancies may be less worried about using medications in their next pregnancy, if they have experienced having a healthy infant despite medication use in a previous pregnancy. Women with chronic diseases or somatic symptoms could have a lower threshold for using analgesics due to a larger total disease burden or more experience with drug treatment in pregnancy. In order to identify women at risk for suboptimal migraine treatment during pregnancy, we need more information on factors affecting drug use in pregnancy, as the literature is inconsistent.

## Conclusions

Many women using analgesics for headache or migraine stop or switch to paracetamol when they become pregnant. A considerable proportion of women with migraine in pregnancy report high headache intensity, and the majority of these are not taking analgesics or just taking paracetamol. These findings are important for clinical practice, as they may contribute to a better understanding of how pregnant women handle their migraine. Counselling on safe analgesic use in pregnancy, with focus on adequate pain relief, should be endeavored for all women of childbearing age suffering from migraine or headache.

## Additional files

Additional file 1: Table S1. Comparison of maternal characteristics in the Akershus Birth Cohort (all participants and study sample) and the Medical Birth Registry of Norway (MBRN)*. (DOCX 15 kb)

Additional file 2: Figure S1. Analgesic use before and during pregnancy. (PDF 113 kb)

Additional file 3: Figure S2. Analgesic use among women with migraine in pregnancy and high headache intensity. (PDF 109 kb)

## Abbreviations

ABC study: Akershus Birth Cohort Study
Ahus: Akershus University Hospital
ATC: Anatomical Therapeutic Chemical
EPDS: Edinburgh Postnatal Depression Scale
MBRN: Medical Birth Registry of Norway
NSAIDs: Non-steroidal anti-inflammatory drugs
OR: Odds ratio
OTC: Over-the-counter
PRIME-MD: Primary care Evaluation on Mental Disorders
Q1: Questionnaire 1 (gestational week 17)
Q2: Questionnaire 2 (gestational week 32)
Q3: Questionnaire 3 (8 weeks after delivery)
ROC: Receiver Operating Characteristics
SCL: Symptoms Checklist
