# Correction to: Patterns and predictors of analgesic use in pregnancy: a longitudinal drug utilization study with special focus on women with migraine

**DOI:** 10.1186/s12884-022-04525-1

**Published:** 2022-04-08

**Authors:** Gerd-Marie Eskerud Harris, Mollie Wood, Malin Eberhard-Gran, Christofer Lundqvist, Hedvig Marie Egeland Nordeng

**Affiliations:** 1 Pharmacoepidemiology & Drug Safety Research Group, Department of Pharmacy, School of Pharmacy, Faculty of Mathematics and Natural Sciences, University of Oslo, P.O. Box. 1068, Blindern, 0316 Oslo, Norway; 2 Department of Child Health, National Institute of Public Health, Oslo, Norway; 3 Health Services Research, Research Department, Akershus University Hospital and University of Oslo, Campus Ahus, Lørenskog, Norway

**Correction to: BMC Pregnancy Childbirth 17, 224 (2017)**

**https://doi.org/10.1186/s12884-017-1399-0**

In Fig. 3, the names on the y-axis on the two bottom graphs were switched. The correct names are “Use of triptans” on the left bottom graph, and “Use of opioids” on the right bottom graph. The corrected Figure is provided below. This error has no implications for the interpretation of the findings.

**Fig. 3:**
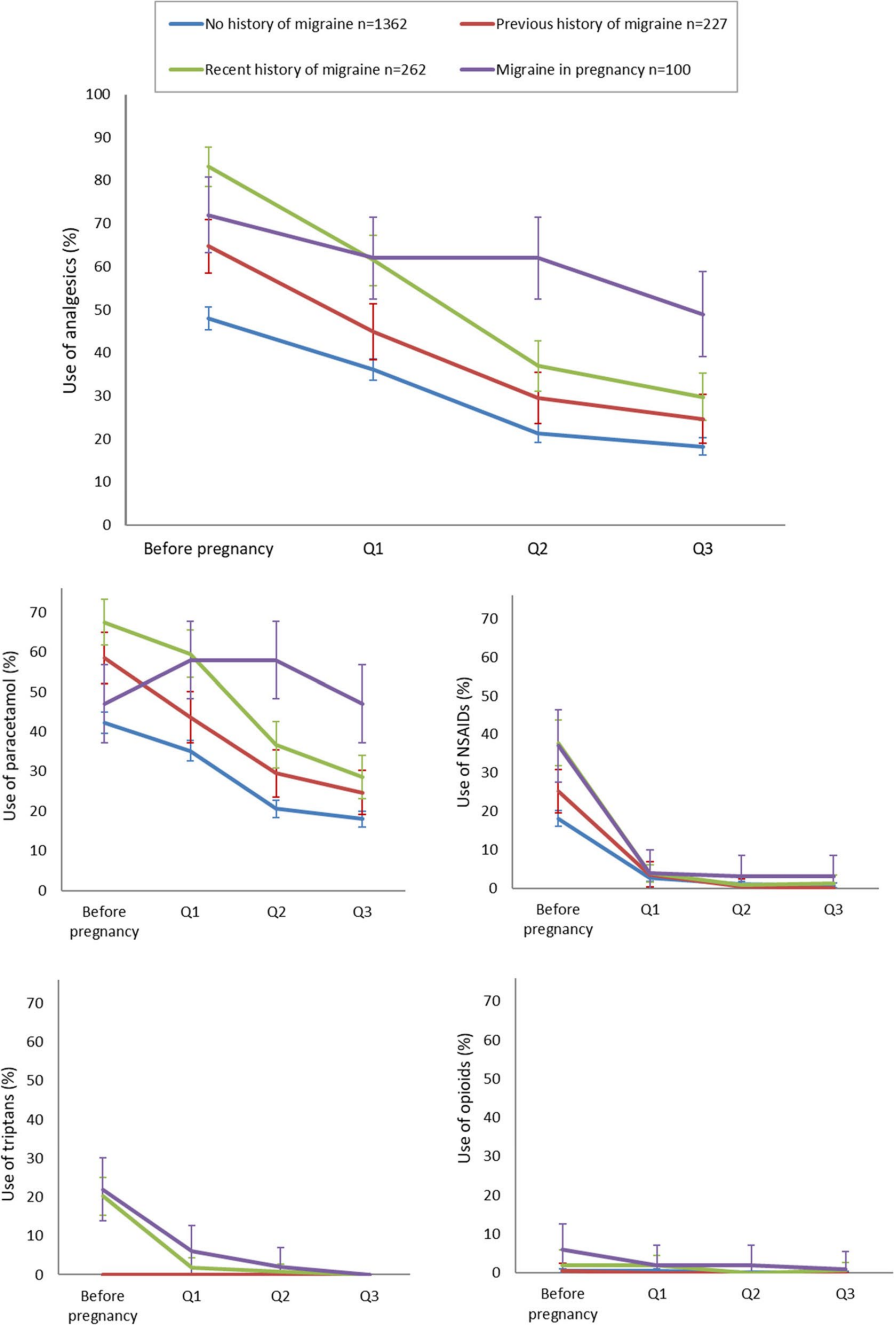
Patterns of total analgesic use and use of specific analgesics before and during pregnancy among women with no migraine history, previous history of migraine, recent history of migraine (within 1 year prior to pregnancy) and migraine in the past month (% users in each group with 95% confidence intervals). Analgesics include paracetamol, NSAIDs, opioids and triptans, used for either migraine or headache

