# Supplementary material for: Patterns and predictors of analgesic use in pregnancy: a longitudinal drug utilization study with special focus on women with migraine
Source: BMC Pregnancy Childbirth. 2017 Jul 14;17:224. doi: 10.1186/s12884-017-1399-0 (PMC5512742; doi:10.1186/s12884-017-1399-0)
Supplement: Supplementary file 1 — Comparison of maternal characteristics in the Akershus Birth Cohort (all participants and study sample) and the Medical Birth Registry of Norway (MBRN)*. (DOCX 15 kb) [file 12884_2017_1399_MOESM1_ESM.docx]

| **Additional file 1: Table S1.** Comparison of maternal characteristics in the Akershus Birth Cohort (all participants and study sample) and the Medical Birth Registry of Norway (MBRN)* | | | |
| --- | --- | --- | --- |
|  | **Full cohort**  **n=4623 (%)** | **Study sample**  **n=1981 (%)** | **MBRN**  **%** |
| Maternal age at delivery |  |  |  |
| *<25* | 12.6 | 8.3 | - |
| *25-30* | 29.8 | 29.7 | - |
| *31-35* | 33.9 | 38.8 | - |
| *>35* | 19.5 | 22.5 | - |
| *Mean age* | 30.7 years | 31.3 years | 30.6 years |
| Parity |  |  |  |
| *First time mother* | - | 49.9 | 42.9 |
| *≥1 previous child* | - | 50.1 | 57.1 |
| Marital status |  |  |  |
| *Married/cohabiting* | 91.3 | 96.4 | 92.3 |
| *Single/divorced/separated* | 3.7 | 2.2 | 7.7 |
| Education |  |  |  |
| *College/university* | 54.7 | 65.3 | - |
| *Primary/secondary school* | 37.7 | 30.6 | - |
| Smoking at time of delivery |  |  |  |
| *No* | 86.1 | 92.9 | 92.6 |
| *Yes* | 6.6 | 3.8 | 7.4 |
| Chronic diseases |  |  |  |
| *None* | 38.4 | 41.9 | - |
| *1 disease* | 37.4 | 37.7 | - |
| *≥ 2 diseases* | 20.1 | 19.8 | - |
| Pregnancy related diseases |  |  |  |
| *None* | 85.4 | 89.7 | - |
| *≥ 1 disease* | 10.4 | 9.6 | - |
| Headache intensity |  |  |  |
| *Low* | 28.3 | 37.4 | - |
| *Moderate* | 37.6 | 49.4 | - |
| *High* | 10.4 | 9.2 | - |
| *Mean headache intensity* | 4.1 | 3.8 | - |
| *data from 2010, accessed from the Medical Birth Registry of Norway, web page of the Institute of Public Health 2016. Maternal characteristics based on information in one or two questionnaires are not included. Numbers do not add up to the total in each group due to missing values: maternal age full cohort 4.3% and study sample 0.7%, marital status full cohort 5.0% and study sample 1.4%, education full cohort 7.7% and study sample 4.1%, smoking full cohort 7.3% and 3.2%, chronic diseases full cohort 4.1% and study sample 0.6%, pregnancy related diseases full cohort 4.2% and study sample 0.7%, headache intensity full cohort 23.7% and study sample 3.9%. | | | |
