# Supplementary figures and images for: Patterns and predictors of analgesic use in pregnancy: a longitudinal drug utilization study with special focus on women with migraine
Source: BMC Pregnancy Childbirth. 2017 Jul 14;17:224. doi: 10.1186/s12884-017-1399-0 (PMC5512742; doi:10.1186/s12884-017-1399-0)

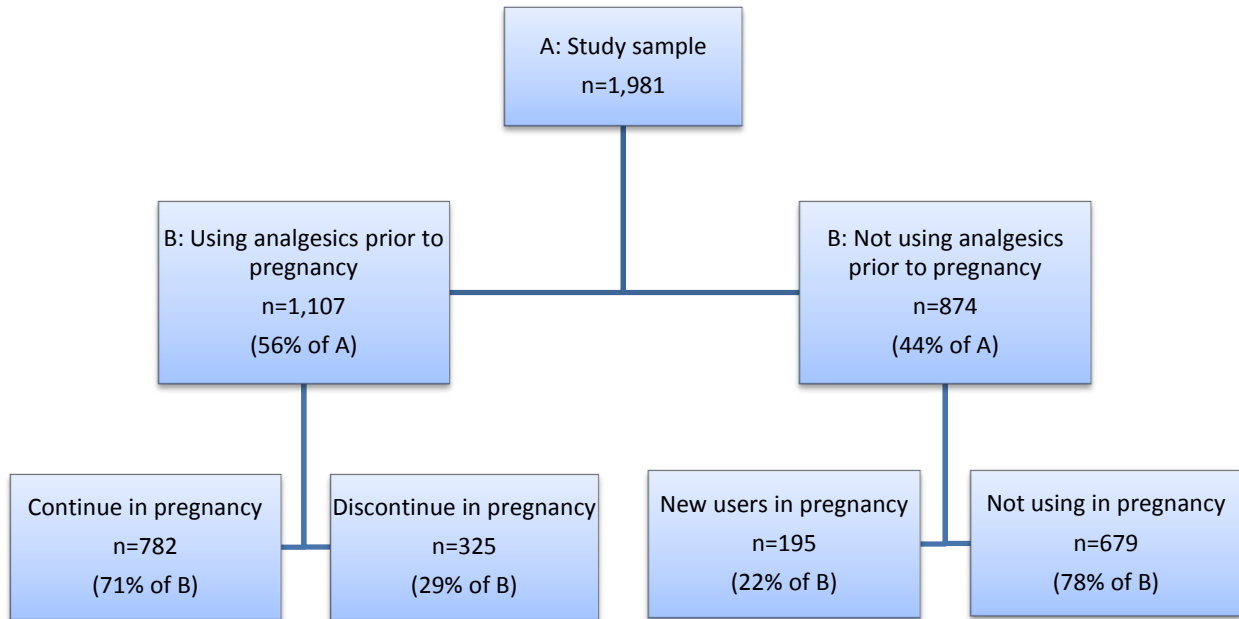

Supplement: Supplementary file 2 — Analgesic use before and during pregnancy. (PDF 113 kb) [file 12884_2017_1399_MOESM2_ESM.pdf]
