# Supplementary material for: Patterns and predictors of analgesic use in pregnancy: a longitudinal drug utilization study with special focus on women with migraine
Source: BMC Pregnancy Childbirth. 2017 Jul 14;17:224. doi: 10.1186/s12884-017-1399-0 (PMC5512742; doi:10.1186/s12884-017-1399-0)
Supplement: Supplementary file 3 — Analgesic use among women with migraine in pregnancy and high headache intensity. (PDF 109 kb) [file 12884_2017_1399_MOESM3_ESM.pdf]

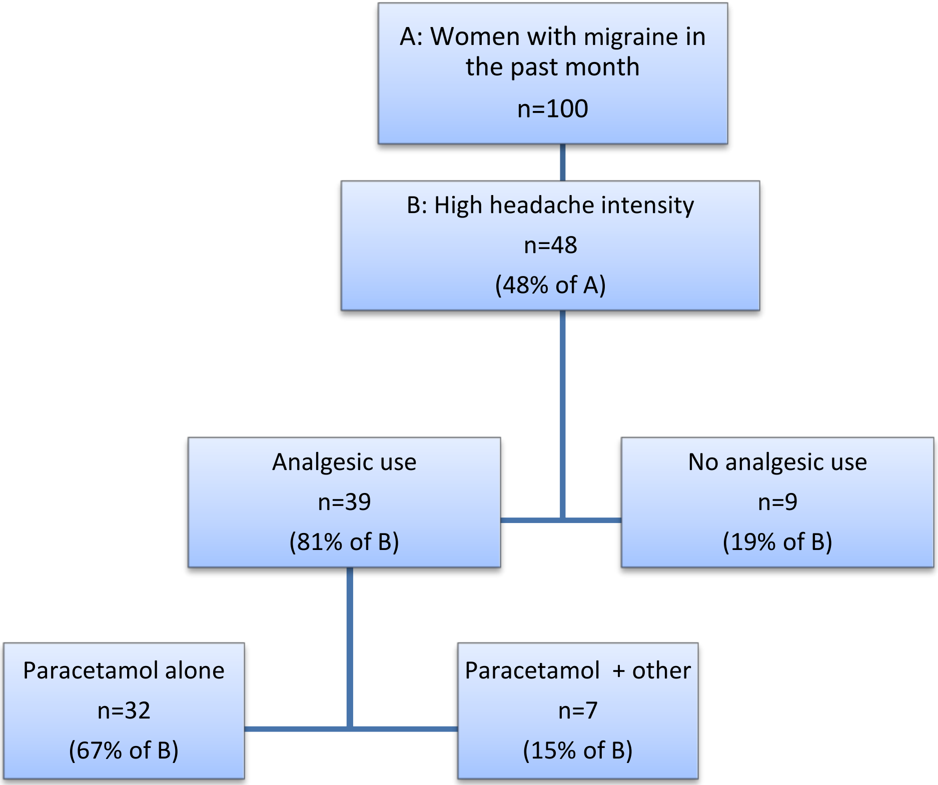
A: Women with migraine in the past month
n=100
B: High headache intensity
n=48
(48% of A)
Analgesic use
n=39
(81% of B)
No analgesic use
n=9
(19% of B)
Paracetamol alone
n=32
(67% of B)
Paracetamol + other
n=7
(15% of B)
